# Higher Uptake of Preoperative 11C-Methionine Positron Emission Tomography Related to Preoperative Seizure in Patients With Oligodendroglioma

**DOI:** 10.7759/cureus.76991

**Published:** 2025-01-06

**Authors:** Madan Bajagain, Akihisa Sakamoto, Tomoko Takajo, Ryutaro Makino, Hiroyuki Uchida, Keisuke Masuda, Nayuta Higa, Hajime Yonezawa, Kazutaka Yatsushiro, Akihide Tanimoto, Ryosuke Hanaya

**Affiliations:** 1 Department of Neurosurgery, Graduate School of Medical and Dental Sciences, Kagoshima University, Kagoshima, JPN; 2 Department of Neurosurgery, Fujimoto General Hospital, Miyakonojo, JPN; 3 Department of Pathology, Graduate School of Medical and Dental Sciences, Kagoshima University, Kagoshima, JPN

**Keywords:** 1p/19q codeletion, astrocytoma, epilepsy, methionine pet, oligodendroglioma

## Abstract

Objective: Epileptic seizures are common in patients with low-grade gliomas (LGGs). ^11^C-methionine (MET) is a radiolabeled amino acid tracer commonly used in positron emission tomography (PET), and MET-PET is a valuable tool for the clinical characterization of gliomas. This study aimed to evaluate the factors associated with MET uptake in patients with suspected LGG and, specifically, to characterize MET uptake in the presence or absence of epilepsy.

Methods: MET uptake on preoperative MET-PET images was retrospectively reviewed in 40 patients with a presurgical diagnosis of LGG based on the absence of gadolinium enhancement on MRI. All patients underwent surgery and had their pathological diagnosis confirmed. The correlation between MET uptake and the occurrence of seizures in patients with LGGs was investigated during their clinical course. The ratio of the lesion to the contralateral normal region (L/N ratio) was calculated by dividing the maximum standardized uptake value of the tumor by the value of the contralateral region. Clinical parameters and MET uptake data were extracted from the medical records.

Results: The mean age of the patients was 48 years (range: 21-90 years), consisting of 26 males and 14 females. Preoperative seizures and 1p/19q codeletion were correlated with higher MET uptake (p < 0.01). Grade 2 oligodendrogliomas had significantly higher MET uptake than grade 2 astrocytomas and glioblastomas (p < 0.01). In particular, grade 2 oligodendrogliomas with preoperative seizures showed approximately twofold higher MET uptake than those without preoperative seizures (L/N ratio 2.01 vs. 1.20, p = 0.042). The optimal cutoff value of the lesion-to-contralateral-normal ratio of MET uptake for predicting preoperative seizures was 2.13, as calculated using a receiver operating characteristic curve and an area under the curve. Conversely, grade 2 astrocytoma and glioblastoma with or without seizures (L/N ratio 1.44 vs. 1.09, 1.40 vs. 1.34), as well as oligodendroglioma without seizures (L/N ratio 1.33), showed similar MET uptake.

Conclusions: An epileptogenic formation mechanism may be involved in the increased uptake of MET in oligodendrogliomas. In patients with suspected LGGs, if there is a significant increase in MET uptake, the possibility of an oligodendroglioma should be considered and attention should be paid to the risk of epileptic seizures.

## Introduction

Epileptic seizures represent the most prevalent early manifestation of low-grade gliomas (LGG), with an estimated 65%-90% of patients experiencing seizures at the initial stages of disease onset [1,2]. LGGs are more likely to cause epilepsy when confined to the cortex of the frontal or the temporal lobes [3]. The mechanism of epileptogenesis in patients with glioma is complex and influenced by multiple factors, including alterations in the peritumoral microenvironment [4,5], glutamate-induced cortical hyperexcitability [6,7], and altered intracellular chloride and gamma-aminobutyric acid activities [8,9]. Additionally, mutations in isocitrate dehydrogenase (IDH)-1 [10-12], p53, and O-6-methylguanine-DNA methyltransferase (MGMT) [13] have been identified as contributing factors. Moreover, the prevalence of seizures varies considerably depending on the pathological type, location, and grade of tumors [2,14]. Epilepsy significantly impacts the overall quality of life of patients with glioma, underscoring the importance of early risk assessment.

^11^C-methionine (MET) positron emission tomography (PET) is a valuable tool for grading and differentiating gliomas [15-17], identifying biopsy targets [18,19], prognosticating preoperative gliomas [15,17,20], detecting glioma recurrence [17], differentiating gliomas from radiation necrosis [21,22], and planning for radiotherapy [23,24]. Several studies have employed MET-PET to investigate the occurrence of seizures in patients with LGGs prior to surgical intervention [1,25]. However, no correlation has been identified between preoperative MET-PET intensity and the occurrence of seizures in all LGGs or astrocytoma cases with IDH mutants. In contrast, previous reports have indicated that patients with drug-resistant epilepsy caused by focal cortical dysplasia (FCD) exhibit higher MET uptake [26,27]. Glioneural tumors, such as dysembryoplastic neuroepithelial tumors and gangliogliomas, exhibit higher MET uptake than those with FCD in pediatric patients with epilepsy [28]. These findings suggest a correlation between higher MET uptake observed on MET-PET for identifying epileptic foci and seizure occurrence. Oligodendrogliomas often complicate epilepsy [1,3] and tend to have higher MET uptake [16]. However, the underlying mechanism remains unclear. In this study, the clinical data of patients with LGGs who underwent preoperative MET-PET were analyzed to determine whether a correlation exists between preoperative seizure occurrence and MET uptake.

## Materials and methods

Patient selection and study design

The inclusion criteria for this retrospective study were as follows: (1) adult patients aged 20 years or older, (2) a presurgical diagnosis of LGG characterized by the absence of gadolinium enhancement on MRI, and (3) a postoperative pathological diagnosis based on the 2021 World Health Organization (WHO) Classification of Tumours of the Central Nervous System [29]. The exclusion criteria included the following: (1) a history of prior treatment for glioma, such as surgery, radiotherapy, or chemotherapy; (2) a diagnosis of high-grade glioma indicated by gadolinium enhancement on MRI during the initial consultation; (3) evidence of other malignancies confirmed via MRI; and (4) unavailability of MET-PET data. Out of 72 eligible patients, 40 who underwent MET-PET imaging for glioma diagnosis and surgical planning between 2003 and 2020 were included in the study. MET-PET imaging was performed at Fujimoto General Hospital. The study period was determined by the availability of MET-PET imaging at Fujimoto General Hospital and the necessary time frame for validating histological diagnoses through clinical follow-up. Among the participants, 34 patients underwent tumor resection via craniotomy, while six underwent biopsy procedures. Resected specimens were subjected to histopathological examination following surgical intervention.

All tumors were evaluated for IDH-1 mutation status and 1p/19q codeletion status. IDH-1 mutation was evaluated using immunohistochemistry anti-Human IDH-1 R132H (dianova, Eching, Germany) in dilution of 1:20. Fluorescence in situ hybridization for 1p/19q codeletion was performed using Vysis LSI 1p36/LSI 1q25 and LSI 19q13/LST 19p13 Dual-Color Probe (Abbott Molecular Inc., Illinois, USA). Based on IDH-1 mutation and 1p/19q codeletion status, tumors were classified as astrocytoma (IDH*-*mutant), oligodendroglioma (IDH*-*mutant, 1p19q codeletion), and glioblastoma (IDH*-*wild type). The number of frozen tumor sections was insufficient to evaluate *CDKN2A/B* homozygous deletion in older cases. Therefore, we performed an immunohistochemical evaluation of methylthioadenosine phosphorylase (MTAP) expression on paraffin-embedded sections as a surrogate marker for *CDKN2A* homozygous deletion to classify astrocytomas into grades 2, 3, and 4. MTAP was evaluated using MTAP monoclonal antibody (M01), clone 2G4 (Abnova, Taiwan) in dilution of 1:200. This approach was based on previous studies [30,31]. Clinical data were extracted from medical and examination records, and seizures were determined based on clinical observations. The correlations among preoperative seizure events, MET uptake, and other clinical parameters were examined. This study was approved by the Ethics Committee of Kagoshima University Hospital (reference no. 170073). The requirement for informed consent was waived owing to the retrospective and noninvasive nature of the study. All eligible patients or their parents were offered the opportunity to opt out of the study.

MET-PET and MRI

The MET tracer was administered intravenously at a dose of 185-280 mBq (5-7.5 mCi). The summed activity 20 min after tracer injection was used for image reconstruction. The images were stored in 128 × 128 × 16 anisotropic voxels with a voxel size of 1 × 1 × 2.6 mm. The PET images were interpreted independently by two experienced nuclear physicians who were blinded to the clinical and anatomical imaging findings, and a consensus was reached. The tracer uptake by the lesion was evaluated using both visual and semiquantitative analyses. A semiquantitative analysis was conducted by placing a region of interest (ROI) over the entire lesion on a transverse PET image, and the standardized uptake value (SUV) was calculated. For lesions exhibiting reduced or equivalent tracer uptake, the ROI was delineated in accordance with anatomical data pertaining to brain lesions derived from MRI or computed tomography. The normal ROI was delineated on the contralateral side of tumors of an equivalent size. The ratio of the lesion to the contralateral normal region (L/N ratio) was calculated by dividing the maximum SUV (SUVmax) of the tumor by that of the contralateral region.

MRI was conducted using a 1.5- or 3-Tesla system at either Kagoshima University Hospital or Fujimoto General Hospital. Glioma volume was estimated using axial T2-weighted and fluid-attenuated inversion recovery images. To avoid discrepancies between MET metabolism and MRI findings, the MRI and MET-PET images were co-registered using Elements (BrainLab, Munich, Germany).

Statistical analysis

The statistical analyses were conducted using GraphPad Prism 9 software (MDF Co., Ltd., Tokyo, Japan). A multiple regression analysis was used to evaluate the correlation between the L/N ratio and other parameters, including age, sex, location (frontal), tumor volume, preoperative seizure, IDH-1mutation, 1p/19q codeletion, and MIB-1 index in all samples and grade 2 gliomas. A paired t-test was used to ascertain any significant differences in the L/N ratio among the various tumor types. The receiver operating characteristic (ROC) curve was used to assess the diagnostic accuracy of MET-PET, with the results expressed as the area under the ROC curve (AUC). The optimal cutoff values for PET scans were identified to maximize sensitivity and specificity in distinguishing WHO grade 2 tumors with and without preoperative seizures. Statistical significance was set at p < 0.05.

## Results

Patient profile

**Figure 1:**
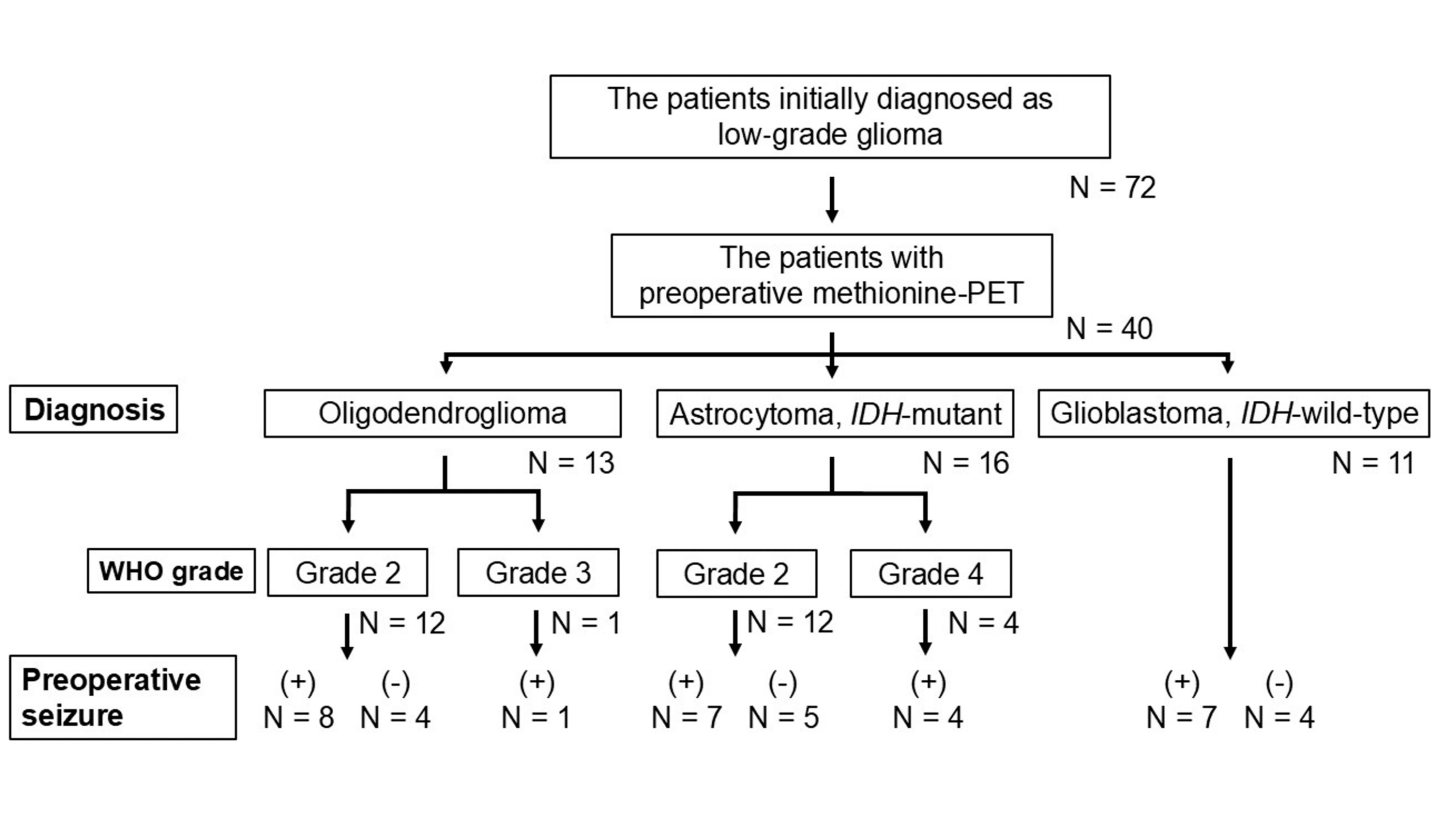
Diagnostic flow for patients with low-grade glioma who were enrolled in this study IDH: isocitrate dehydrogenase; PET: positron emission tomography; N: number; (+): with preoperative seizure; (-): without preoperative seizure The presurgical diagnosis of low-grade glioma was made based on nongadolinium enhancement on MRI

Figure 1 illustrates the process of selecting and diagnosing the 40 patients with suspected LGGs. Twelve patients were diagnosed with astrocytoma (IDH-mutant), WHO grade 2 (grade 2 astrocytoma), and four patients were diagnosed with astrocytoma (IDH-mutant), WHO grade 4 (grade 4 astrocytoma). Eleven patients were diagnosed with glioblastoma (IDH-wild type), WHO grade 4. Thirteen patients were diagnosed with oligodendroglioma (IDH-mutant, 1p19q codeletion), whereas 12 were identified as having WHO grade 2 (grade 2 oligodendroglioma). One patient was diagnosed with a grade 3 oligodendroglioma.

Table 1 presents the patient profiles. Twenty-seven of the 40 patients (67.5%) experienced seizures prior to surgical intervention. The majority of gliomas were located in the frontal lobe (67.5%), with cortical involvement observed in all cases. Gliomas were predominantly confined to the left hemisphere in 24 patients, accounting for 60% of all cases. Of the 27 patients who experienced epileptic seizures prior to surgery, 16 presented with generalized seizures (likely focal to bilateral tonic-clonic seizures), five with focal impaired awareness seizures, and six with focal aware seizures. All patients with preoperative seizures were administered antiseizure medications after the occurrence of seizures, and prior to surgery, each patient exhibited optimal seizure control. The remaining 13 patients who did not experience seizures did not receive prophylactic antiseizure medications prior to surgical intervention. The mean age at the time of the PET study of glioma was 47.5 years.

**Table 1:** Demographic information of the patients enrolled in this study L/N ratio: The ratio of the lesion and contralateral normal region; mean ± standard deviation. #All seizures were considered to be focal to bilateral tonic-clonic seizures, as they were to be caused by the tumor

	Values (number, % of total value)		
	Total	With preoperative seizure	Without preoperative seizure
Total number of patients	40	27 (67.5%)	13 (32.5%)
Age (years)			
Average and SD	46.9 ± 15.9	47.5 ± 15.2	45.7 ± 15.9
Median and range	48 (21-90)	47.5 (26-90)	49 (21-71)
Gender			
Male	26	19 (73.1%)	7 (26.9%)
Female	14	8 (57.1%)	6 (42.9%)
(Male/female ratio)	1.86/1	2.38/1	1.17/1
Tumor location			
Frontal lobe	27	17 (63.0%)	10 (37.0%)
Parietal lobe	3	3 (100%)	0
Temporal lobe	1	1 (100%)	0
Insular lobe	1	0	1 (100%)
Occipital lobe	0	0	0
Multilobar	8	7 (87.5%)	1 (12.5%)
Right hemisphere	16	11 (68.8%)	5 (31.2%)
Left hemisphere	24	16 (66.7%)	8 (33.3%)
Tumor volume (m^3^)			
Average and SD	34.4 ± 14.3	49.5 ± 28.1	36.3 ± 34.3
Median and range	35.6 (9.0-87.6)	43 (10.2-93.5)	23 (9.0-130.0)
Type of seizure			
Generalized seizure #		16	
Focal impaired awareness seizure		5	
Focal aware seizure		6	
Methionine accumulation (L/N ratio)			
Average and SD	1.65 ± 0.63	1.84 ± 0.67	1.24 ± 0.25
Median and Range	1.46 (1.00-3.41)	1.73 (1.00-3.41)	1.15 (1.00-1.67)
Pathological diagnosis			
Oligodendroglioma			
Grade 3	1	1 (100%)	0 (0.0%)
Grade 2	12	8 (66.7%)	4 (33.3%)
Astrocytoma,IDH-mutant			
Grade 4	4	4 (100%)	0 (0.0%)
Grade 2	12	7 (58.3%)	5 (41.7%)
Astrocytoma, IDH-wild type	11	7 (63.6%)	4 (36.4%)
Mib-1 index			
Average and SD	4.6 ± 3.5	4.4 ± 3.5	5.0 ± 3.8
Median and range	3.0 (1.0-15.0)	3.0 (1.0-15.0)	4.0 (1.0-13.0)

Preoperative MET uptake and preoperative seizure occurrence

Multiple regression analyses of all cases revealed a strong association between preoperative seizures, 1p/19q codeletion (oligodendroglioma), and an increased L/N ratio (increased MET uptake) (Table 2).

**Table 2:** Factors associated with preoperative MET uptake in patients diagnosed with low-grade glioma based on preoperative MRI findings: results of multiple regression analysis SE: standard error; **: p < 0.01 Multivariate regression analysis was performed to evaluate the relationship between methionine uptake and clinical variables in patients diagnosed with low-grade gliomas based on preoperative MRI findings. Statistical tests included t-tests for regression estimates

Variable	Estimate	95% confidence interval	SE	t	p-value
Age	0.0004	-0.012-0.013	0.006	0.062	0.951
Sex	-0.1708	-0.574-0.232	0.198	-0.865	0.394
Location (frontal)	-0.0528	-0.515-0.410	0.227	-0.233	0.817
Tumor volume	0.0032	-0.004-0.010	0.003	0.955	0.347
Preoperative seizure	0.4976	0.133-0.862	0.179	2.785	0.009**
IDH-1 mutation	0.0414	-0.444-0.527	0.238	0.174	0.863
1p/19q codeletion	0.7245	0.291-1.158	0.213	3.406	0.002**
MIB-1 index	-0.0002	-0.051-0.051	0.025	-0.008	0.994

**Figure 2:**
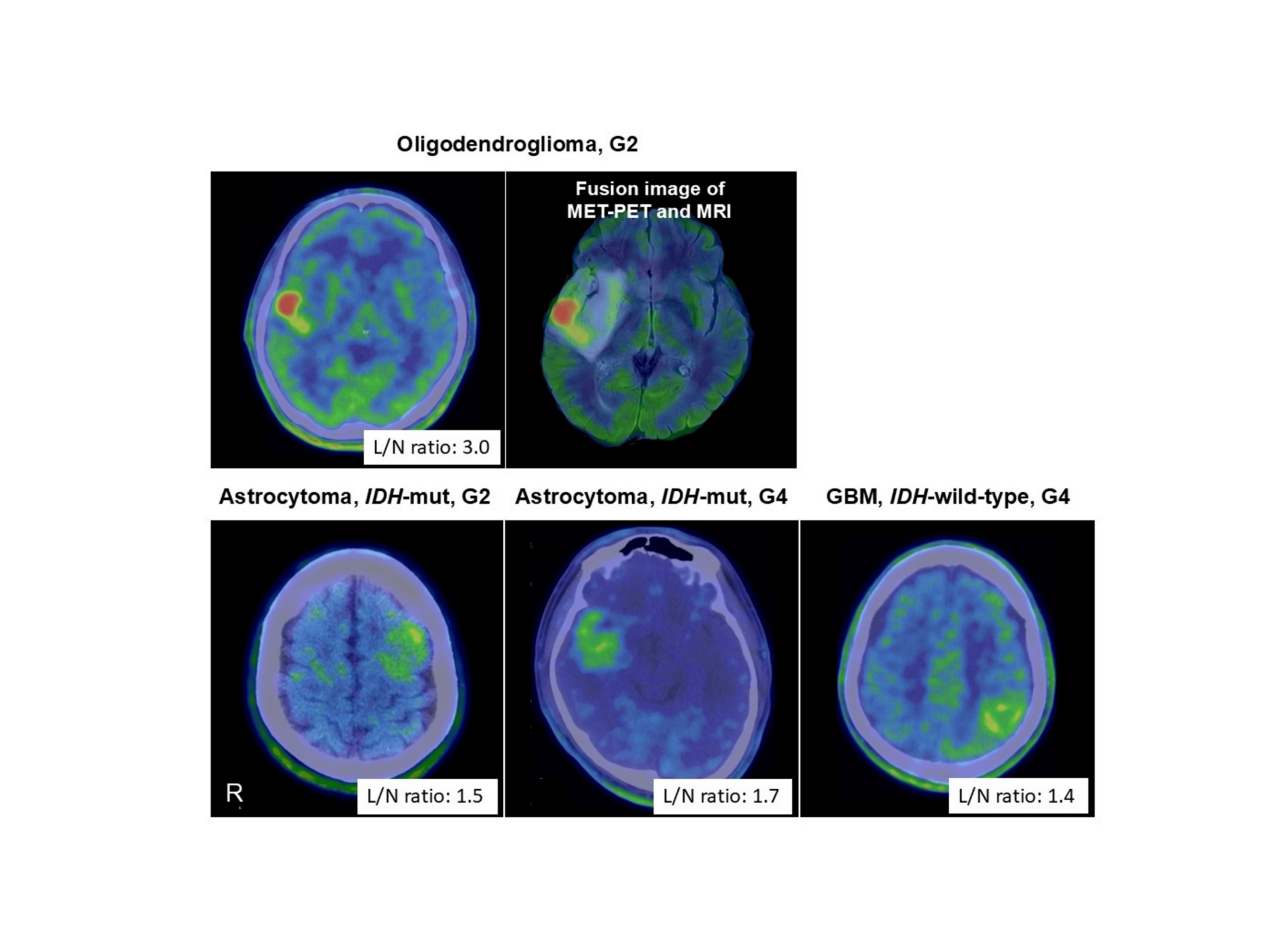
Typical preoperative methionine-PET findings in each tumor type with preoperative seizures G2: World Health Organization grade 2; G4: World Health Organization grade 4; GBM: glioblastoma; IDH: isocitrate dehydrogenase; L/N ratio: the lesion to contralateral normal region ratio; mut: mutant; R: right The sites of methionine accumulation were assessed using fusion images of MET-PET and MRI-FLAIR. In gliomas without gadolinium enhancement on MRI, oligodendrogliomas associated with preoperative seizures demonstrated higher methionine uptake

**Figure 3:**
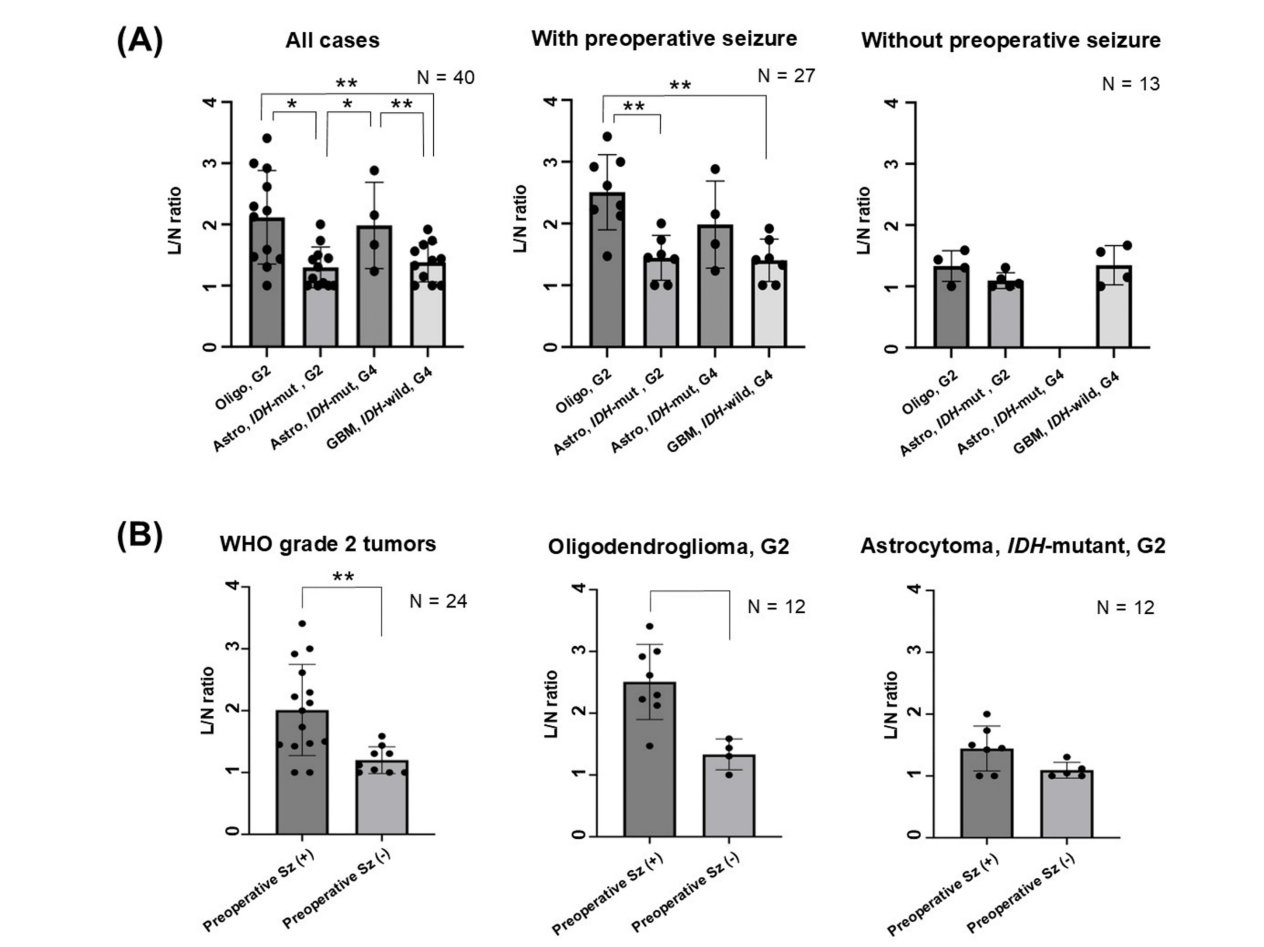
Relationship between the presence and absence of preoperative seizures and methionine uptake Astro: astrocytoma; G2: World Health Organization grade 2; G4: World Health Organization grade 4; GBM: glioblastoma; IDH: isocitrate dehydrogenase; L/N ratio: the lesion to contralateral normal region ratio; mut: mutant; Oligo: oligodendroglioma; R: right; Sz: seizure; Sz (+): with seizure; Sz (-): without seizure; WHO: World Health Organization. * p < 0.05; ** p < 0.01 (A) Association between tumor type and methionine (MET) uptake in all enrolled cases. MET uptake (L/N ratio) was significantly higher in grade 2 oligodendroglioma and grade 4 astrocytoma than in glioblastoma and grade 2 astrocytoma when all cases were included. Additionally, in cases where seizures occurred prior to surgery, grade 2 oligodendroglioma showed a significantly higher MET uptake (L/N ratio) than glioblastoma or grade 2 astrocytoma. In cases without presurgical seizures, no such difference was observed. (B) Relationship between the presence and absence of preoperative seizures and MET uptake in WHO grade 2 tumors. In grade 2 oligodendrogliomas, MET uptake (L/N ratio) was significantly increased in cases with preoperative seizures

The sole case of grade 3 oligodendroglioma (L/N ratio: 1.87) was excluded from the comparison between tumor groups. MET accumulation was higher in grade 2 oligodendrogliomas and grade 4 astrocytomas than in glioblastomas and grade 2 astrocytomas across all 39 cases (Figures 2-3A). Moreover, a comparison between groups with and without epilepsy-related seizures revealed that grade 2 oligodendrogliomas and grade 4 astrocytomas exhibited elevated MET uptake compared to grade 2 astrocytomas and glioblastomas. However, no significant differences were observed among various tumors in patients without preoperative seizures.

Similarly, multiple regression analysis of grade 2 astrocytomas and oligodendrogliomas revealed that preoperative seizures and 1p/19q codeletion (oligodendroglioma) were significantly associated with an increase in the L/N ratio (reflecting increased MET uptake) (Table 3).

**Table 3:** Factors associated with preoperative MET uptake in patients diagnosed with WHO grade 2 astrocytoma and oligodendroglioma: results of multiple regression analysis MET: 11C-methionine; SE: standard error; **: p < 0.01 Multiple regression analysis was performed to evaluate the relationship between methionine uptake and clinical variables in WHO grade 2 astrocytoma and oligodendroglioma. Statistical tests included t-tests for regression estimates

Variable	Estimate	95% confidence interval	SE	t	p-value
Age	0.011	-0.007-0.029	0.008	1.324	0.204
Sex	-0.371	-0.890-0.149	0.245	-1.511	0.150
Location (frontal)	0.246	-0.348-0.841	0.280	0.880	0.392
Tumor volume	0.003	-0.007-0.012	0.004	0.614	0.548
Preoperative seizure	0.586	0.130-1.042	0.215	2.727	0.014*
1p/19q codeletion	0.894	0.418-1.370	0.224	3.985	0.001*
MIB-1 index	0.018	-0.052-0.088	0.033	0.535	0.600

A comparison of patients with and without preoperative seizures revealed that patients with seizures exhibited higher MET uptake than those without seizures for all grade 2 tumors (L/N ratio 2.01 vs. 1.20, p = 0.042; Figure 3B). Furthermore, patients with grade 2 astrocytomas who experienced seizures exhibited a higher mean L/N ratio than patients without seizures; however, this difference was not statistically significant (p = 0.682). In contrast, oligodendrogliomas with seizures exhibited significantly higher MET uptake than those without seizures (p = 0.0045). The diagnostic efficacy of preoperative seizures based on the L/N ratio in grade 2 oligodendrogliomas was considerable (AUC, 0.969; 95% confidence interval: 0.882-1.000), with a cutoff value of 2.125 (sensitivity, 0.875; specificity, 1.00).

## Discussion

Role of MET-PET in preoperative diagnosis of suspected LGGs

MET uptake by brain tumors is influenced by several factors, including amino acid transport, MET metabolism, tumor vascular bed-dependent blood flow, microvascular density, and distribution across the blood-brain barrier [32-34]. Accordingly, MET uptake has been shown to be increased in patients with high-grade glioma [16].

In our study, patients with suspected high-grade glioma with gadolinium enhancement on MRI had been excluded; however, among all patients predicted to have LGGs before surgery, 11 were diagnosed with glioblastoma, which was also included. Our study showed that grade 4 astrocytoma had a higher increase in MET uptake compared to glioblastoma and grade 2 astrocytoma. However, there was no significant difference in MET accumulation between glioblastomas and grade 2 astrocytomas. No findings of necrosis and/or microvascular proliferation were observed in any grade 4 gliomas.

Most studies on MET-PET findings in gliomas have been performed according to the conventional classification of brain tumors. Further genetic testing, including alpha-thalassemia/mental retardation, X-linked (ATRX), telomerase reverse transcriptase promoter (TERTp), and O-6-methylguanine-DNA methyltransferase (MGMT), would be beneficial but was difficult to conduct in this study. Further evaluation of MET-PET findings in astrocytomas and glioblastomas is required.

Relationship between increased MET uptake in oligodendroglioma and epileptic seizures

In the present study, there was no significant correlation between preoperative MET uptake and tumor proliferative capacity (Mib-1 index) in patients with LGGs and grade 4 gliomas. This finding is consistent with previous studies that reported a high L/N ratio does not reflect glioma proliferation [16,35,36], as observed in grade 2 oligodendrogliomas with seizures.

The mechanisms underlying epileptogenesis in tumors are not completely understood and are thought to be multifactorial. These mechanisms can be broadly classified as due to the direct effects of the tumor (tumor centric) or changes in the extracellular environment that cause cortical hyperexcitability (epilepsy centric) [5,37]. The molecular characteristics of gliomas, particularly IDH mutations, are thought to be one of the causes of high MET uptake in patients with LGGs [14]. Additionally, it has been reported that gliomas with IDH-1/-2 mutations are more likely to cause epileptic seizures than IDH-1/-2-wild-type tumors [38]. The mutated IDH reduces α-ketoglutarate to D-2-hydroxyglutarate, which has a structure similar to glutamate and can simulate the activity of glutamate in N-Methyl-D-aspartate receptors, potentially leading to seizures [10,38]. This study showed differences in the incidence of preoperative seizures between grade 2 and grade 4 astrocytomas in the IDH mutation group (58.3% vs. 100%, respectively). The incidence of preoperative seizures in patients with glioblastomas was 63.6%, which was comparable to that in patients with grade 2 astrocytomas. Although the number of cases was limited, this finding supports the view that the IDH mutation is not a decisive factor in the development of epilepsy in gliomas [39].

Prior to the 2021 WHO classification, oligodendrogliomas were known to have a higher MET uptake than diffuse astrocytomas [16]. In the present study, based on the 2021 classification, MET uptake was higher in grade 2 oligodendrogliomas than in grade 2 astrocytomas. In other words, at least in the presurgical stage, it is suggested that 1p/19q co-deletion exerts the greatest influence on the increase in MET uptake in grade 2 gliomas. It has been reported that there is no significant correlation between MET-PET and the occurrence of epilepsy in grade 2 gliomas [1]. The same study showed an L/N ratio of 2.2 for the astrocytoma/oligoastrocytoma group and 2.5 for the oligodendroglioma group. According to the WHO 2021 classification, oligoastrocytoma is now diagnosed as oligodendroglioma, and the findings may differ if the diagnosis is based on the current tumor classification. Our findings revealed that grade 2 oligodendrogliomas with epileptic seizures exhibited significantly higher MET uptake (L/N ratio: 2.51 ± 0.61) compared to other tumor types, including molecular glioblastomas, when analyzed based on the presence or absence of epilepsy. The MET uptake of grade 2 oligodendrogliomas without epileptic seizures was equivalent to that observed in other tumor types (L/N ratio: 1.33 ± 0.25).

Enhanced MET uptake has also been reported in focal cortical dysplasia with epilepsy [26,27]. It is possible that enhanced MET uptake plays a role in the mechanisms underlying the development of epilepsy. In addition, there is an overlap between the mechanisms of epileptogenic region formation and tumor growth [39]. The incidence of oligodendroglioma seizures is approximately 75% at initial diagnosis [40]. In epilepsy, MET uptake increases approximately twofold. These findings suggest that the previously reported higher MET uptake in oligodendrogliomas may be attributed to epileptogenic formation.

Limitations

This study had several limitations. Due to the retrospective nature of this study, not all eligible patients underwent MET-PET during the inclusion period, and 40 of 72 (56%) patients with LGG underwent MET-PET. As a result, the sample size was relatively small, and the number of PET scans for each tumor type was also small; therefore, selection bias cannot be ruled out. Variations in the MRI examination protocols, including differences in the timing of scans, equipment, sequences, and types of gadolinium-based contrast agents used across facilities, resulted in nonuniform conditions. Furthermore, it was challenging to evaluate the impact of antiepileptic drugs on methionine uptake. In addition, this study included patients up to 2020 to confirm the diagnoses based on their clinical courses. To comply with the 2021 WHO Classification of Tumours based on molecular diagnosis, additional tests were performed on samples from these patients where necessary. There is a possibility that this may have caused differences in the power of the test in some cases. Similarly, it is possible that some cases classified as IDH-wild type (glioblastoma) should have been categorized as astrocytomas since only IDH-1 mutations were evaluated and IDH-2 mutations were not assessed. Therefore, a prospective study based on molecular diagnosis with a larger sample size is required to exclude these factors.

## Conclusions

This study showed that an increase in preoperative MET uptake was associated with the occurrence of preoperative epileptic seizures in LGGs, especially oligodendrogliomas. This may be a useful indicator to consider in the prophylactic administration of anticonvulsants in patients suspected of having LGGs without surgical intervention. Additionally, in the absence of seizures, MET-PET has also been shown to be unable to differentiate between glioblastomas, grade 2 astrocytomas, and oligodendrogliomas in patients suspected of having LGGs. Further advances in noninvasive methods for diagnosing gliomas are required.

## References

[1] Danfors T, Ribom D, Berntsson SG, Smits A (2009). Epileptic seizures and survival in early disease of grade 2 gliomas. Eur J Neurol.

[2] Pallud J, Audureau E, Blonski M (2014). Epileptic seizures in diffuse low-grade gliomas in adults. Brain.

[3] Goldstein ED, Feyissa AM (2018). Brain tumor related-epilepsy. Neurol Neurochir Pol.

[4] Armstrong TS, Grant R, Gilbert MR, Lee JW, Norden AD (2016). Epilepsy in glioma patients: mechanisms, management, and impact of anticonvulsant therapy. Neuro Oncol.

[5] Pallud J, Capelle L, Huberfeld G (2013). Tumoral epileptogenicity: how does it happen?. Epilepsia.

[6] Stephens ML, Williamson A, Deel ME (2014). Tonic glutamate in CA1 of aging rats correlates with phasic glutamate dysregulation during seizure. Epilepsia.

[7] Neal A, Yuen T, Bjorksten AR, Kwan P, O'Brien TJ, Morokoff A (2016). Peritumoural glutamate correlates with post-operative seizures in supratentorial gliomas. J Neurooncol.

[8] Pallud J, Le Van Quyen M, Bielle F (2014). Cortical GABAergic excitation contributes to epileptic activities around human glioma. Sci Transl Med.

[9] MacKenzie G, O'Toole KK, Moss SJ, Maguire J (2016). Compromised GABAergic inhibition contributes to tumor-associated epilepsy. Epilepsy Res.

[10] Chen H, Judkins J, Thomas C (2017). Mutant IDH1 and seizures in patients with glioma. Neurology.

[11] Duan WC, Wang L, Li K (2018). IDH mutations but not TERTp mutations are associated with seizures in lower-grade gliomas. Medicine (Baltimore).

[12] Yang Y, Mao Q, Wang X, Liu Y, Mao Y, Zhou Q, Luo J (2016). An analysis of 170 glioma patients and systematic review to investigate the association between IDH-1 mutations and preoperative glioma-related epilepsy. J Clin Neurosci.

[13] Feyissa AM, Worrell GA, Tatum WO (2019). Potential influence of IDH1 mutation and MGMT gene promoter methylation on glioma-related preoperative seizures and postoperative seizure control. Seizure.

[14] Samudra N, Zacharias T, Plitt A, Lega B, Pan E (2019). Seizures in glioma patients: an overview of incidence, etiology, and therapies. J Neurol Sci.

[15] Singhal T, Narayanan TK, Jacobs MP, Bal C, Mantil JC (2012). 11C-methionine PET for grading and prognostication in gliomas: a comparison study with 18F-FDG PET and contrast enhancement on MRI. J Nucl Med.

[16] Shinozaki N, Uchino Y, Yoshikawa K, Matsutani T, Hasegawa A, Saeki N, Iwadate Y (2011). Discrimination between low-grade oligodendrogliomas and diffuse astrocytoma with the aid of 11C-methionine positron emission tomography. J Neurosurg.

[17] Nariai T, Tanaka Y, Wakimoto H (2005). Usefulness of L-[methyl-11C] methionine-positron emission tomography as a biological monitoring tool in the treatment of glioma. J Neurosurg.

[18] Massager N, David P, Goldman S (2000). Combined magnetic resonance imaging- and positron emission tomography-guided stereotactic biopsy in brainstem mass lesions: diagnostic yield in a series of 30 patients. J Neurosurg.

[19] Pirotte B, Goldman S, Massager N (2004). Comparison of 18F-FDG and 11C-methionine for PET-guided stereotactic brain biopsy of gliomas. J Nucl Med.

[20] Riva M, Lopci E, Castellano A (2019). Lower grade gliomas: relationships between metabolic and structural imaging with grading and molecular factors. World Neurosurg.

[21] Terakawa Y, Tsuyuguchi N, Iwai Y, Yamanaka K, Higashiyama S, Takami T, Ohata K (2008). Diagnostic accuracy of 11C-methionine PET for differentiation of recurrent brain tumors from radiation necrosis after radiotherapy. J Nucl Med.

[22] Tsuyuguchi N, Sunada I, Iwai Y (2003). Methionine positron emission tomography of recurrent metastatic brain tumor and radiation necrosis after stereotactic radiosurgery: is a differential diagnosis possible?. J Neurosurg.

[23] Navarria P, Reggiori G, Pessina F (2014). Investigation on the role of integrated PET/MRI for target volume definition and radiotherapy planning in patients with high grade glioma. Radiother Oncol.

[24] Langen KJ, Galldiks N, Hattingen E, Shah NJ (2017). Advances in neuro-oncology imaging. Nat Rev Neurol.

[25] Bono BC, Ninatti G, Riva M (2024). The role of preoperative [11C]methionine PET in defining tumor-related epilepsy and predicting short-term postoperative seizure control in temporal lobe low-grade gliomas. Neurosurg Focus.

[26] Madakasira PV, Simkins R, Narayanan T, Dunigan K, Poelstra RJ, Mantil J (2002). Cortical dysplasia localized by [11C]methionine positron emission tomography: case report. Am J Neuroradiol.

[27] Sasaki M, Kuwabara Y, Yoshida T (1998). Carbon-11-methionine PET in focal cortical dysplasia: a comparison with fluorine-18-FDG PET and technetium-99-ECD SPECT. J Nucl Med.

[28] Phi JH, Paeng JC, Lee HS (2010). Evaluation of focal cortical dysplasia and mixed neuronal and glial tumors in pediatric epilepsy patients using 18F-FDG and 11C-methionine pet. J Nucl Med.

[29] (2021). Central Nervous System Tumours. WHO Classification of Tumours, 5th Edition. https://publications.iarc.fr/Book-And-Report-Series/Who-Classification-Of-Tumours/Central-Nervous-System-Tumours-2021.

[30] Satomi K, Ohno M, Matsushita Y (2021). Utility of methylthioadenosine phosphorylase immunohistochemical deficiency as a surrogate for CDKN2A homozygous deletion in the assessment of adult-type infiltrating astrocytoma. Mod Pathol.

[31] Gundogdu F, Babaoglu B, Soylemezoglu F (2024). Reliability assessment of methylthioadenosine phosphorylase immunohistochemistry as a surrogate biomarker for CDKN2A homozygous deletion in adult-type IDH-mutant diffuse gliomas. J Neuropathol Exp Neurol.

[32] van Waarde A, Elsinga PH (2008). Proliferation markers for the differential diagnosis of tumor and inflammation. Curr Pharm Des.

[33] Ishiwata K, Kubota K, Murakami M, Kubota R, Sasaki T, Ishii S, Senda M (1993). Re-evaluation of amino acid PET studies: can the protein synthesis rates in brain and tumor tissues be measured in vivo?. J Nucl Med.

[34] Omidi Y, Barar J (2012). Impacts of blood-brain barrier in drug delivery and targeting of brain tumors. Bioimpacts.

[35] Kato T, Shinoda J, Oka N (2008). Analysis of 11C-methionine uptake in low-grade gliomas and correlation with proliferative activity. AJNR Am J Neuroradiol.

[36] van der Meer PB, Taphoorn MJ, Koekkoek JA (2022). Management of epilepsy in brain tumor patients. Curr Opin Oncol.

[37] Nowell M, Miserocchi A, McEvoy AW (2015). Tumors in epilepsy. Semin Neurol.

[38] Ohno M, Hayashi Y, Aikawa H (2021). Tissue 2-hydroxyglutarate and preoperative seizures in patients with diffuse gliomas. Neurology.

[39] de Jong JM, Broekaart DW, Bongaarts A, Mühlebner A, Mills JD, van Vliet EA, Aronica E (2022). Altered extracellular matrix as an alternative risk factor for epileptogenicity in brain tumors. Biomedicines.

[40] Mirsattari SM, Chong JJ, Hammond RR, Megyesi JF, Macdonald DR, Lee DH, Cairncross JG (2011). Do epileptic seizures predict outcome in patients with oligodendroglioma?. Epilepsy Res.

